# A Case of Hepatitis C-Related Decompensated Cirrhosis Observed by MRI Imaging Data During Treatment With Direct-Acting Antiviral Agents

**DOI:** 10.7759/cureus.19001

**Published:** 2021-10-24

**Authors:** Atsushi Nakamura, Tsubasa Yoshimura, Takeshi Ichikawa

**Affiliations:** 1 Hepatology, Nippon Koukan Hospital, Kawasaki, JPN; 2 Gastroenterology, Nippon Koukan Hospital, Kawasaki, JPN

**Keywords:** protein energy malnutrition (pem), magnetic resonance imaging-proton density fat fraction, magnetic resonance elastography, hepatitis c, decompensated cirrhosis

## Abstract

Sofosbuvir/velpatasvir therapy can safely treat hepatitis C virus (HCV)-related decompensated cirrhosis and has been shown to improve liver function at an early stage. However, the pathophysiology of the liver during treatment remains unclear. In this case report, we analyzed hepatic morphology on magnetic resonance imaging during the treatment period and confirmed that liver function and malnutrition were greatly improved with the elimination of HCV, and that rapid hemodynamic changes were occurring in the liver.

## Introduction

For a long time, liver transplantation had been the only treatment for patients with hepatitis C virus (HCV)-related decompensated cirrhosis. With the recent approval of sofosbuvir (SOF)/velpatasvir (VEL) therapy, a direct-acting antiviral (DAA) therapy, decompensated cirrhosis can now be treated safely and effectively, and successful treatment has been shown to result in early improvement in liver function [1]. However, the pathophysiological changes during treatment in decompensated cirrhosis, as well as the long-term benefits of DAA therapy remain unclear.

In this paper, we report the changes in liver stiffness using magnetic resonance elastography (MRE) and portal hemodynamics before, during, and at the end of treatment (EOT) in a case of decompensated cirrhosis treated with SOF/VEL for 12 weeks. In addition, we evaluated the short-term improvement of malnutrition in this case using magnetic resonance imaging (MRI) data analysis.

## Case presentation

**Figure 1:**
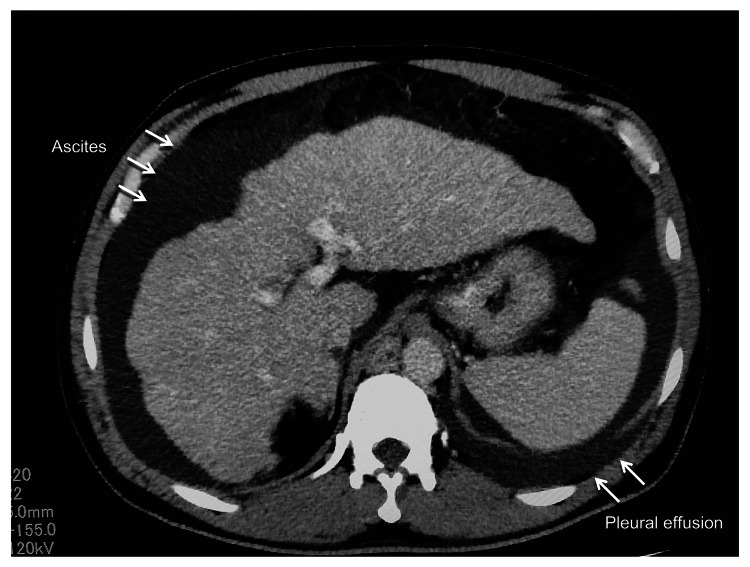
CT imaging findings CT, computed tomography

A 51-year-old man with HCV infection (serotype 1) who had previously failed DAA treatment due to decompensated cirrhosis was admitted to the Nippon Koukan Hospital with a complaint of abdominal distension. He had no history of HCV treatment, no history of HBV infection, and had not been drinking for a year. He was 174 cm tall, weighed 86.2 kg, and was in a clear state of consciousness. Blood tests showed serum total bilirubin 2.4 mg/dL, aspartate aminotransferase (AST) 81 IU/L, alanine aminotransferase (ALT) 53 IU/L, alkaline phosphatase 300 IU/L, γ-glutamyl transpeptidase 47 IU/L, white blood cell count 4210/μL, hemoglobin 17.2 g/dL, platelet count 4.9 × 10^4^/μL, and HCV RNA level 6.1 log IU/mL. Liver function was severely impaired, with a prothrombin activity of 53.1% and a blood ammonia level of 163 μg/dL, which was class C in the Child-Pugh classification. Albumin was 2.7 g/dL, total cholesterol 80 mg/dL, and triglyceride 63 mg/dL, suggesting protein energy malnutrition. Computed tomography (CT) and MRI showed moderate ascites and left-sided pleural effusion (Figure 1), but no hepatocellular carcinoma was noted. The liver stiffness on MRE was 5.719 kPa, which is consistent with cirrhosis, and the MRI-proton density fat fraction (PDFF) was 2.7%, showing a low tendency. Esophagogastroduodenoscopy (EGD) showed F2 grade esophageal varices.

**Figure 2:**
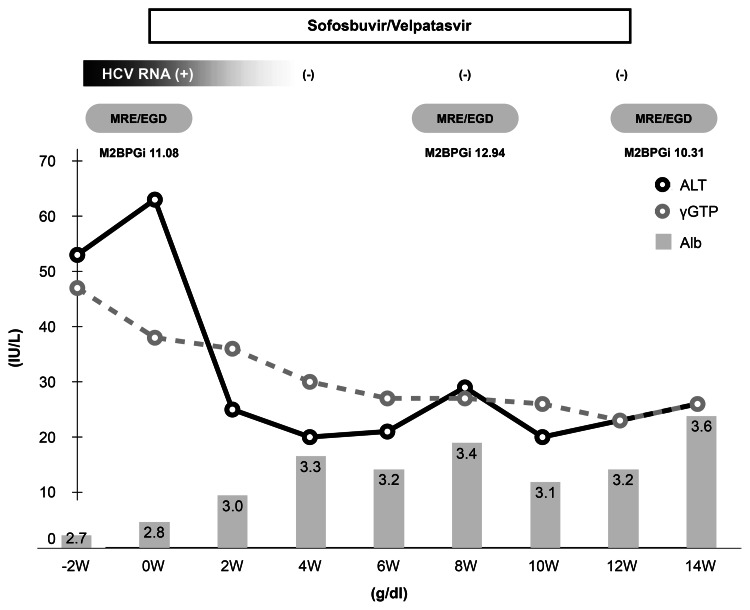
The clinical course of the present case HCV, hepatitis C virus; ALT, alanine aminotransferase; γGTP, γ-glutamyl transpeptidase; Alb, albumin

We started furosemide and spironolactone for ascites in cirrhosis and lactulose for hyperammonemia. Since this patient had progressed to decompensated cirrhosis, we administered 12 weeks of SOF/VEL treatment, and, after informed consent, the patient agreed to this treatment plan. The patient was treated with endoscopic variceal ligation to prevent the rupture of esophageal varices, and then antiviral therapy was started (Figure 2).

**Figure 3:**
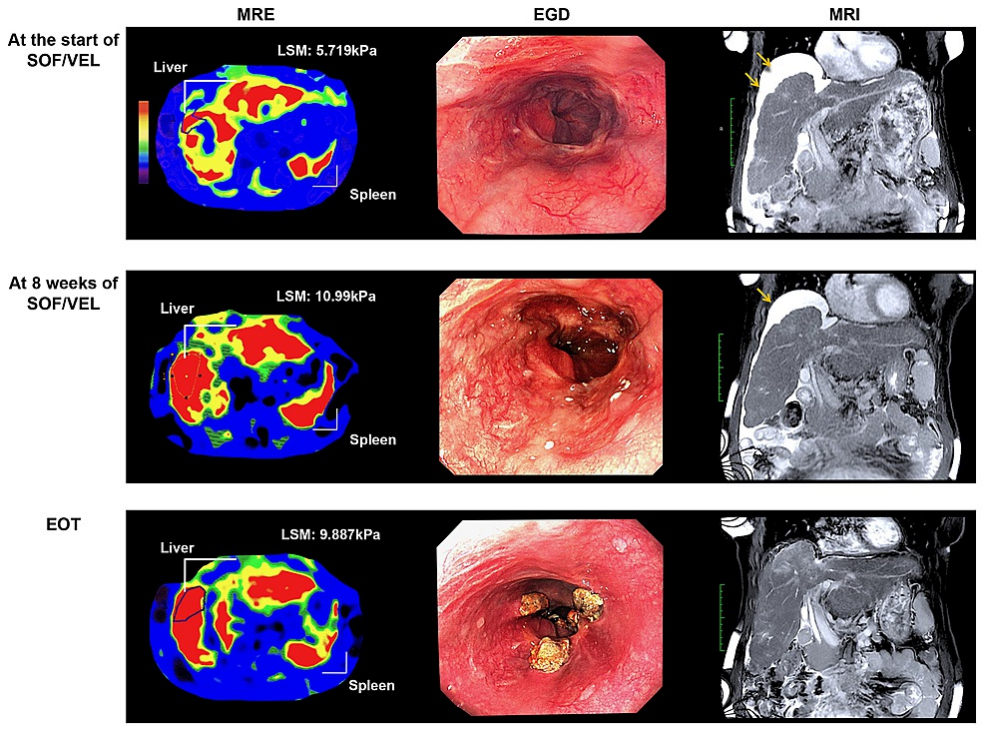
MRI and EGD images before, during, and at the end of treatment Images of liver and esophageal varices before SOF/VEL therapy (top row), after eight weeks of therapy (middle row), and at the end of therapy (bottom row). From left to right: MRE, EGD, and MRI of coronal sections. EGD images at the end of therapy are after re-treatment of esophageal varices. MRE shows the color mapping of the shear stiffness (kPa) of the organ, which changes from blue to red as the stiffness increases. In the present case, liver stiffness increased markedly to 10.99 kPa at eight weeks of treatment. The color mapping of the spleen also suggested an increase in stiffness. SOF, sofosbuvir; VEL, velpatasvir; MRE, magnetic resonance elastography; EGD, esophagogastroduodenoscopy; MRI, magnetic resonance imaging

With the initiation of SOF/VEL, his AST and ALT levels decreased rapidly, and his serum HCV RNA was undetectable four weeks after the start of treatment and remained negative until EOT. His albumin level also increased from the early stage of SOF/VEL treatment, reaching 3.6 g/dL at EOT, and ascites almost completely disappeared. We were concerned about the recurrence of esophageal varices and the worsening of ascites during DAA treatment, so we performed imaging studies eight weeks after the start of SOF/VEL treatment (Figure 3).

**Figure 4:**
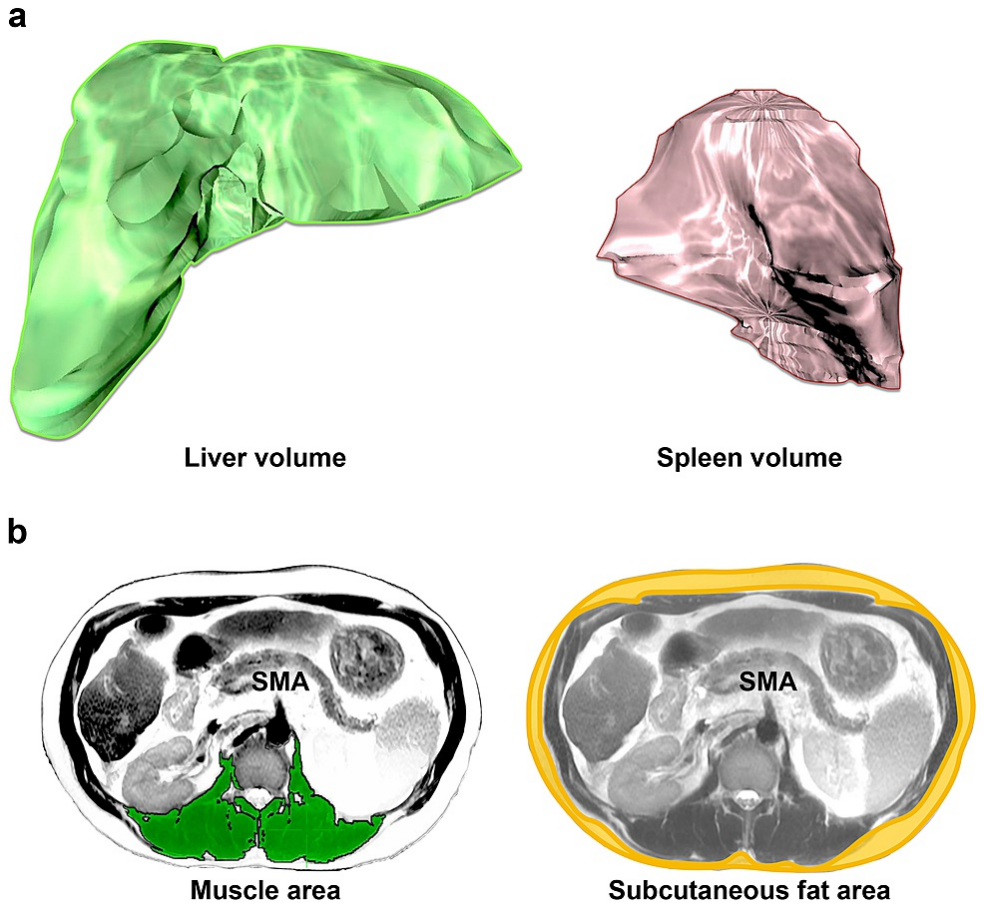
Each measurement method using digital image data from MRI Digital MRI data in this case was analyzed using OsiriX version 6.0 open-source software. (a) Liver volume and splenic volume were measured. (b) Dorsal muscle area and subcutaneous fat area were measured at the level of the SMA. The measurement data are shown in Table 1. MRI, magnetic resonance imaging; SMA, superior mesenteric artery

Ascites remained, EGD showed a re-increase in the size of the treated varices, and a marked increase in liver stiffness was found on MRE measurement. And, serum mac-2 binding protein glycosylation isomer (M2BPGi), a marker of liver fibrosis, was also elevated from baseline (Figure 2). Due to the patient’s work schedule, the re-treatment of the esophageal varices with EGD was performed the week after the EOT. The endoscopic findings at that time showed a slight reduction of esophageal varices dilation and a decrease in liver stiffness by MRE measurement. Analysis of the MRI digital data using OsiriX version 6.0 open-source software (64-bit, Pixmeo, Geneva, Switzerland; http://www.osirix-viewer.com) showed a marked increase in splenic volume (mL) at eight weeks after the start of treatment and a slight increase in hepatic volume (mL) in the late stage of treatment. In addition, body weight (kg), MRI-PDFF (%), and subcutaneous fat area (cm^2^) and muscle area (cm^2^) measured using MRI imaging increased as compared with pretreatment measurements, suggesting an improvement in nutritional status (Figure 4, Table 1).

**Table 1:** Each measurement from the MRI digital image data is shown (liver volume, spleen volume, liver fat content, subcutaneous fat mass, and muscle mass) Values indicate % increase from baseline MRI, magnetic resonance imaging; PDFF, proton density fat fraction; SFA, subcutaneous fat area; SOF, sofosbuvir; VEL, velpatasvir

	Liver volume (mL)	Spleen volume (mL)	MRI-PDFF (%)	SFA (cm^2^)	Muscle area (cm^2^)
At the start of SOF/VEL	2030	511	2.7	66	55
At 8 weeks of SOF/VEL	2066 (2%)	611 (20%)	6.5 (141%)	84 (28%)	64 (16%)
At the end of SOF/VEL	2140 (5%)	665 (30%)	8.8 (226%)	85 (29%)	70 (27%)

## Discussion

In a study of SOF/VEL treatment based on Japanese real-world data [2], sustained virologic response (SVR) 12, meaning undetectable serum HCV RNA 12 weeks after EOT, was reported to be 90.2% in decompensated cirrhosis. Along with an even higher SVR rate, serum albumin levels were found to be elevated at EOT in patients who achieved SVR12, and further elevated at SVR12. On the other hand, patients with low serum albumin levels (<2.8 g/dL) at baseline did not show an increase in serum albumin levels after EOT, suggesting that baseline albumin levels may be a predictor of liver function recovery after SVR. In the present case, the baseline Child-Pugh classification was class C, and the albumin level was low at 2.7-2.8 g/dL. However, after the start of SOF/VEL administration, AST and ALT rapidly decreased, and albumin quickly increased, improving to 3.6 g/dL at EOT. Wake et al. [3] investigated liver function improvement and changes in liver volume (ratio to standard liver volume) in chronic HCV infections that achieved SVR with DAA therapy. The results showed that liver volume increased significantly only in non-cirrhotic patients and that the increase in albumin levels was significantly correlated with a decrease in ALT levels. Di Maira et al. [4] also conducted a study of DAA-treated patients with HCV-related cirrhosis who were eligible for liver transplantation and reported that baseline liver volume was an independent factor contributing to improved Child-Pugh scores after SVR. These results indicate that the recovery of liver functions, such as albumin, by DAA treatment in cirrhosis is not due to the regenerative effect of hepatocytes but rather to the recovery of remaining hepatocyte functions due to the elimination of inflammation. In the present case, the ratio of liver volume at baseline to standard liver volume was 1.5, and no liver atrophy was observed. Therefore, liver function appears to have recovered rapidly in this case as the inflammation disappeared with the elimination of HCV.

MRI-PDFF is a noninvasive and highly accurate method of measuring intrahepatic fat content. In the current case, MRI-PDFF significantly increased at eight weeks after the start of SOF/VEL treatment and increased threefold from the baseline value at EOT. Chronic HCV can cause hepatic steatosis due to decreased secretion of very low-density lipoproteins caused by apolipoprotein abnormalities, promotion of bile acid synthesis, and suppression of bile acid degradation [5]. Tada et al. [6] reported that SVR with DAA treatment of chronic HCV significantly reduced MRI-PDFF, but our case showed the opposite result. A study of 2349 healthy civilians reported [7] a median MRI-PDFF of 4% and a 25th percentile value of 2.7%; therefore, the baseline MRI-PDFF of this patient was low, which thus indicated a state of hepatic fat loss. Therefore, we interpreted the increase in MRI-PDFF in this case as the recovery of hepatic fat mass due to the improvement of liver function caused by the elimination of HCV. As Enooku et al. [8] have demonstrated, the decreased expression of fatty acid transport protein-5 in hepatocytes is associated with hepatic fat loss in advanced non-alcoholic steatohepatitis. In addition, early increases in body fat and muscle mass as measured by MRI were also observed. Fatty acids are the largest stored energy source in the body, and the liver acts as a central hub for fatty acid metabolism. Magnetic resonance spectroscopy assessments of liver transplant donors have shown a significant positive correlation between liver fat content and subcutaneous fat area, visceral fat area, and trunk muscle area measured by CT [9].^ ^In cirrhosis, Child-Pugh class progression and free fatty acid concentrations in the blood are positively correlated [10]. We speculate that the improvement of fatty acid metabolism in the liver by HCV elimination may have corrected the systemic energy deficiency and improved body composition, but further analysis of more cases is needed.

A notable finding in the present case was a marked increase in liver stiffness at eight weeks after the start of SOF/VEL treatment. At the same time, EGD showed an increase in the size of esophageal varices, which were clearly worsening. MRE also suggested an increase in spleen stiffness. Thus, spleen volume was measured based on MRI analysis and was found to have increased by 20% compared with baseline. Several reports have described the effects of DAA treatment of chronic HCV on changes in liver stiffness and improvement in portal hypertension [11]. In many cases, SVR decreased liver stiffness and portal pressure, but in cases with advanced hepatic fibrosis at baseline, decrease in portal pressure was poor, and in fact, some cases even worsened [12]. Liver fibrosis, inflammation, and congestion have been suggested to contribute to increased liver stiffness [6]. In the present case, AST and ALT decreased rapidly after the start of SOF/VEL treatment, and liver function improved markedly, suggesting that the worsening of inflammation and fibrosis in cirrhosis is unlikely. On the contrary, M2BPGi in the present case showed the same kinetics as that of liver stiffness measured using MRE. A study on M2BPGi in patients with chronic heart failure [13] reported that M2BPGi was elevated in patients with liver dysfunction and decreased in patients with heart failure with improved New York Heart Association functional class. In the present case, no signs of heart failure were observed during the course of treatment. Based on these results, we believe that one of the factors for the marked increase in liver stiffness may be increased intrahepatic vascular resistance due to increased hepatic blood flow. However, we do not have data to verify whether this is an increase in portal blood flow or arterial blood flow, but we speculate that it may be a reversible effect of cirrhosis triggered by HCV elimination. We believe that this case suggests the need for further validation. 

There are some reports of patients with decompensated cirrhosis treated with SOF/VEL who discontinued treatment due to variceal bleeding or liver failure [2].

Based on our experience, DAA treatment in decompensated cirrhosis may improve liver function early on and seem to work well, but portal hypertension may worsen during treatment, especially early on. We recommend that patients with decompensated cirrhosis pay attention to liver stiffness measurements and splenomegaly during DAA treatment.

## Conclusions

We observed the clinical course of patients with decompensated cirrhosis treated with SOF/VEL using MRI data. Significant improvement in liver function and malnutrition was observed with HCV elimination and may be related to hepatocellular function recovery and rapid hemodynamic changes. 
